# Successful Heart Transplantation With Myocardial Bridging: A Case Report on Unroofing Technique

**DOI:** 10.7759/cureus.49165

**Published:** 2023-11-21

**Authors:** Yusuke Tsukioka, Valluvan Jeevanandam

**Affiliations:** 1 Cardiothoracic Surgery, University of Chicago Medicine, Chicago, USA; 2 Cardiac Surgery, University of Chicago Medicine, Chicago, USA

**Keywords:** postoperative cardiac function, coronary artery stenosis, unroofing procedure, heart transplantation, myocardial bridging

## Abstract

Myocardial bridging (MB), a common anatomical variation where a segment of a coronary artery is covered by myocardium, poses a relative contraindication in heart transplantation due to the risk of post-transplant ischemia. This report presents a case of successful transplantation of a donor heart with MB, where unroofing (removal) of the myocardial bridge was performed. The donor was a 42-year-old male with mild nonobstructive coronary artery stenosis and MB. The recipient, a 55-year-old male, suffered from ischemic cardiomyopathy and severe heart failure. During transplantation, unroofing of the donor heart’s MB was executed to mitigate the risk of myocardial ischemia. The transplantation was successful with preserved postoperative cardiac function. The unroofing procedure did not significantly extend ischemic or operative time. Postoperative electrocardiogram (ECG) and echocardiography showed no signs of myocardial ischemia. Donor hearts with MB can be utilized for transplantation with appropriate surgical intervention. This case demonstrates the potential of unroofing procedures in expanding the suitability of donor hearts for transplantation, without increasing the risk of postoperative complications or mortality.

## Introduction

Myocardial bridging (MB) is a normal anatomical variation, defined as a segment of a coronary artery being covered by the myocardium. It is associated with a range of clinical scenarios, from being completely asymptomatic to causing acute pump failure [1-4]. In patients with MB, tachycardia is known to increase the risk of myocardial ischemia. Post-transplant denervated hearts, which tend to develop sinus tachycardia, make the transplantation of donor hearts with MB relatively contraindicated [5]. In this case report, we present a case where unroofing of MB was successfully performed on a donor's heart during transplantation, yielding favorable outcomes, which are illustrated with videos and photographs.

## Case presentation

Donor information

**Figure 1:**
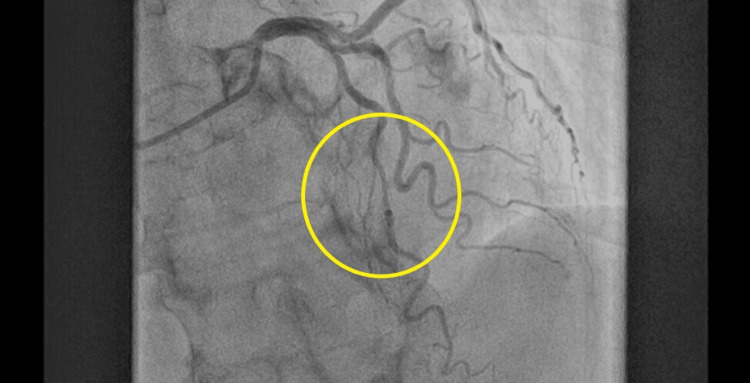
Mildly stenosed LAD due to myocardial bridging Coronary angiography revealed a mild nonobstructive coronary artery stenosis approximately 3 cm in length with myocardial bridging in the mid-LAD. LAD: Left anterior descending artery.

The donor was a 42-year-old male, 178 cm in height and 68 kg in weight (body mass index: 21.5 kg/m^2^), with no significant medical history. He was admitted to the donor hospital, which was near the transplant center, following asphyxiation and was declared brain dead after 14 days. Echocardiography showed good left ventricular function with an ejection fraction (EF) of 60%. Coronary angiography revealed a mild nonobstructive coronary artery stenosis approximately 3 cm in length with MB in the mid-left anterior descending artery (LAD) but was otherwise normal (Figure 1). The donor's electrocardiogram (ECG) was within normal limits, with no signs of myocardial ischemia.

Recipient information

The recipient was a 55-year-old male, 190 cm in height and 101 kg in weight (body mass index: 28.0 kg/m^2^), with a history of coronary artery disease, including myocardial infarction in the LAD territory treated with percutaneous coronary intervention (PCI). His cardiac function was reduced, with an EF of 15%. He was diagnosed with ischemic cardiomyopathy and had undergone cardiac resynchronization therapy with a defibrillator (CRT-D) implantation. Despite aggressive medical therapy, he experienced New York Heart Association (NYHA) class III heart failure symptoms six months before transplantation and was on milrinone infusion under intra-aortic balloon pump (IABP) support preoperatively.

Implantation procedure

During procurement, the donor heart was vented in both the right and left atrium before antegrade perfusion with a University of Wisconsin (UW) cardioplegic solution. The heart was preserved in UW solution at 4°C and transported to our facility. Under general endotracheal anesthesia and in the supine position, the patient was prepped and draped in a standard aseptic manner. A right femoral arterial catheter was placed for monitoring. A median sternotomy was performed. Preparation for cardiopulmonary bypass (CPB) included ACT-guided heparinization, cannulation of the transverse arch under the innominate vein with a 22 French (Fr) cannula (3-0 Surgipro sutures; Covidien, Mansfield, MA), and bicaval cannulation (superior vena cava [SVC], 3-0 Surgipro, 24 Fr DLP; inferior vena cava [IVC], 3-0 Surgipro, 31 Fr DLP). A transverse incision was made in the aorta, and the heart was explanted by transecting the pulmonary artery (PA) and the aorta. The right atrium was removed, and the posterior portion of the left atrium, IVC, and SVC were left for anastomosis. The ascending aorta was resected to the undersurface of the arch. The donor's heart was prepared for transplantation by separating the PA, aorta, SVC, and pulmonary veins.

The MB location was identified based on the coronary angiography images. By palpating the coronary artery, the lesion of the myocardial bridge could be estimated. The adipose tissue was incised with a 10-watt electrocautery (coagulation mode) to expose the muscle. A right-angle clamp was slid between the exposed muscle and the coronary artery, and the muscle was incised with the electrocautery. Care was taken to lift the right-angle clamp sufficiently to avoid damaging the coronary artery during the incision. The cut ends of the myocardium were meticulously cauterized to prevent postoperative bleeding. The unroofing procedure was completed in a duration of merely 4 minutes and 25 seconds (Video 1).

**Video 1:** Unroofing technique for myocardial bridging

A DeVega tricuspid annuloplasty was performed with pledgeted 2-0 Surgipro sutures. The left atrial anastomosis was completed with 3-0 Surgipro sutures. The IVC and SVC (4-0 Surgipro sutures), PA, and aortic (4-0 Surgipro) anastomoses followed. Antegrade cardioplegia was administered into the aortic root after each anastomosis. Warm blood was given antegrade for deairing the coronary arteries. The patient was rewarmed, the pressure was decreased to 50 mmHg, the aortic root suction was applied, and the cross-clamp was released. After deairing the heart, the right atrial incision was closed, and the ascending aorta vent was removed. The rhythm reverted to normal sinus rhythm immediately after the aortic clamp was removed. Cardiac function was excellent. The patient was weaned from CPB with low-dose dopamine and dobutamine. After decannulation and protamine administration, hemostasis was adequate, and 32 Fr chest tubes (posterior/anterior mediastinal, right pleural) were placed. The sternum was closed with wires and plates due to poor sternal condition, immunosuppression, and transverse sternal fractures. A dressing was applied, and the patient was transferred to the intensive care unit in stable condition with normal sinus rhythm. Details of the cardiopulmonary bypass, including duration and flow rates, are comprehensively presented in Table 1.

**Table 1:** Cardiopulmonary bypass data CPB: Cardiopulmonary bypass.

CPB data	
Bypass time	133 minutes
Cross-clamp time	115 minutes
Core temperature	34°C
Flow	5.8 L/min
Implant time	73 minutes
Donor heart ischemic time	134 minutes

Postoperative examination

The ECG showed normal sinus rhythm with no ST elevation. A transesophageal echocardiogram revealed normal left ventricular performance with a left ventricular EF of 60%. No left ventricular regional wall motion abnormalities were noted after the injection of echocardiographic contrast. No valvular abnormalities were identified.

The postoperative course was uneventful, with stable hemodynamics, and the patient was discharged home two weeks postoperatively.

## Discussion

Congestive heart failure remains one of the leading causes of death in the United States, and the need for heart transplantation remains high, despite a shortage of donor hearts. Many transplant centers are expanding their selection criteria to provide heart transplants to more recipients. While donor hearts with MB are considered a relative contraindication for transplantation, reports by Singhal et al. have shown that safe heart transplantation can be performed by releasing the myocardial bridge [5]. Our report is the only case report demonstrating the unroofing technique with video.

The unroofing procedure by Singhal et al. [5] and our team is supported by case reports on MB by Pittaluga et al. [4]. Their report highlighted a case where a donor heart with an asymptomatic myocardial bridge, which did not undergo surgical intervention such as unroofing before transplantation, resulted in infarction at the LAD myocardial bridge site in the recipient, leading to heart failure and death.

In our case, unroofing did not significantly prolong ischemic or operative time. Postoperative cardiac function was preserved, and no ischemic changes were observed on the ECG. Our report demonstrates that donor hearts with MB, which do not compromise cardiac function, can be easily modified and used for transplantation without increasing the risk of postoperative complications or mortality.

The incidence of MB varies depending on the diagnostic method used, generally reported to be less than 5%. However, autopsy studies report incidences ranging from 40% to 50% to as high as 85% [1-4]. These intramuscular arterial segments generally do not exhibit atheromatous arteriosclerosis [1], and atherosclerotic lesions are more commonly found proximal to the myocardial bridge segment [6]. Previous reports have indicated post-transplant myocardial infarction due to occlusion at the site of MB [4], with an incidence rate of approximately 30% in heart transplant recipients [7].

In cases involving young donors, coronary catheterization is not routinely performed. Nevertheless, it is advisable to employ catheterization for the detection of MB.

## Conclusions

We employed a donor heart exhibiting a myocardial bridge in the LAD. MB can cause underperfusion and hemodynamic disturbances in the heart, so unroofing was performed before transplantation. The surgery was completed without significantly extending the operative time, and the recipient showed no postoperative complications. Thus, donor hearts with MB can be surgically modified and used for transplantation without increasing the risk of mortality or complications.
